# Photoactivated Curcumin-Loaded Lipid Nanoparticles in Hydrogel: A Cutting-Edge Intracanal Medicament for Advanced Endodontic Therapy

**DOI:** 10.3390/gels11050308

**Published:** 2025-04-22

**Authors:** Sónia Ferreira, Liliana Grenho, Maria H. Fernandes, Sofia A. Costa Lima

**Affiliations:** 1 Department of Dental Sciences, IUCS-CESPU: University Institute of Health Sciences-Advanced Polytechnic and University Cooperative, CRL, 4585-116 Gandra, Portugal; 2 Laboratório Associado para a Química Verde-Rede de Química e Tecnologia (LAQV, REQUIMTE), Department of Chemical Sciences, Faculty of Pharmacy, Universidade do Porto, Rua de Jorge Viterbo Ferreira 228, 4050-313 Porto, Portugal; 3 BoneLab, Faculdade de Medicina Dentária, Universidade do Porto, Rua Dr. Manuel Pereira da Silva, 4200-393 Porto, Portugal; 4 Laboratório Associado para a Química Verde-Rede de Química e Tecnologia (LAQV, REQUIMTE), Faculdade de Medicina Dentária, Universidade do Porto, Rua Dr. Manuel Pereira da Silva, 4200-393 Porto, Portugal; 5 Laboratório Associado para a Química Verde-Rede de Química e Tecnologia (LAQV, REQUIMTE), Instituto de Ciências Biomédicas de Abel Salazar, Universidade do Porto, Rua de Jorge Viterbo Ferreira 228, 4050-313 Porto, Portugal

**Keywords:** alginate, antimicrobial activity, *Enterococcus faecalis*, rheology, solid lipid nanoparticles, photoactivation

## Abstract

Intracanal reinfections continue to pose a major challenge in endodontic treatment. Photodynamic therapy has emerged as a promising antimicrobial strategy. Regarding this, curcumin (CUR), a natural photosensitizer, shows potential in this context, but its application is hampered by poor solubility and rapid degradation. This study aimed to develop and characterize a CUR-loaded nanoparticle-enriched hydrogel to enhance its stability, sustain its release, and evaluate its antimicrobial efficacy upon photoactivation (PhAc). Curcumin-loaded nanoparticles were synthesized and incorporated into a hydrogel matrix, followed by characterization using scanning electron microscopy, Fourier-transform infrared spectroscopy, in vitro CUR release studies, and rheological analysis. Antibiofilm activity against *Enterococcus faecalis* was assessed under both photoactivated and non-photoactivated conditions. Cytocompatibility was analyzed through fibroblast viability assays and fluorescence staining. The CUR-containing hydrogel demonstrated a sustained release profile extending beyond 72 h. Rheological studies confirmed its shear-thinning behavior, ensuring injectability even after post-photoactivation. Antibiofilm assays revealed a significant reduction in *E. faecalis* biofilms, with PhAc formulations exhibiting markedly enhanced antibacterial efficacy compared to their non-PhAc counterparts. Cytocompatibility assays confirmed that all formulations, including those subjected to PDT, preserved fibroblast viability, indicating biocompatibility suitable for clinical use. In sum, the CUR-containing hydrogel exhibits properties that support its potential as an effective intracanal therapeutic, combining antimicrobial and photodynamic effects to help prevent reinfections in endodontic treatments.

## 1. Introduction

Endodontic treatment aims to achieve optimal disinfection of the root canal system and prevent reinfection, ensuring long-term therapeutic success [1]. Mechanical instrumentation enhances disinfectant penetration, but the complex root canal anatomy and diverse microbial community leave some areas inaccessible to conventional irrigation, making complete disinfection challenging [2]. *Enterococcus faecalis* (*E. faecalis*) is frequently isolated from persistent endodontic infections and treatment failures due to its remarkable adaptability [3]. This pathogenic bacterium stay alive in extreme environments such as high salinity, exposure to detergents, heavy metals, ethanol, and temperatures above 45 °C. Its persistence is linked to its ability to form a biofilm and enter into the dentinal tubule, making it resistant to conventional irrigants and host immune defenses. Additionally, its extracellular polymeric matrix enhances antimicrobial resistance, while proton pump activity helps regulate intracellular pH, contributing to its tolerance to calcium hydroxide, the routinely used compound in endodontic treatment [3,4]. Photodynamic therapy (PDT) is an alternative antimicrobial strategy that generates reactive oxygen species (ROS) through the interaction of a photosensitizing agent, light, and molecular oxygen [5]. These reactive molecules induce oxidative damage, leading to microbial inactivation. A key advantage of PDT is its specific action against microorganisms while preserving host tissues [4]. Research has demonstrated its effectiveness in eliminating local infections through direct bacterial killing and interfering with virulence factors such as biofilm formation and bacterial adhesion in the root canal system [6]. Additionally, some studies suggest that PDT may enhance dentin properties by increasing collagen cross-linking [7,8].

Curcumin (CUR), a bioactive curcuminoid from *Curcuma longa* L., exhibits broad antimicrobial activity by disrupting bacterial cytokinesis, inducing filamentation and inhibiting Z-ring formation [9]. The rise of multidrug resistance has driven the search for new therapeutics, with CUR emerging as a promising natural antimicrobial agent [10]. Its low cytotoxicity, anti-inflammatory properties, and photosensitizing capacity make it a promising candidate for combination therapies, including antimicrobial PDT [11]. As CUR absorbs light between 300 and 500 nm, leading to ROS production, it exhibits strong potential as a photosensitizing agent against antimicrobial PDT [12]. However, its limited water solubility, poor bioavailability, rapid metabolism, and chemical instability hinder its effectiveness as a therapeutic agent. Recent advances in nanotechnology have provided solutions to CUR’s physico-chemical challenges [13,14]. In a previous study, solid lipid nanoparticles (SLNs) composed of cetyl palmitate/Tween^®^ 80 were developed as a delivery system for CUR. These nanoparticles exhibited improved CUR solubility, sustained release properties, and enhanced antimicrobial efficacy towards *E. faecalis* [15].

Hydrogels are promising materials for drug delivery systems due to their fluidity, adaptability, and controlled drug release properties. They are easy to prepare, widely available, and have low toxicity [3]. The encapsulation of nano-photosensitizers within hydrogels provides additional protection, reducing the risk of rapid ROS release and minimizing toxicity [16]. Among biopolymers, alginate is particularly interesting for its natural origin, biocompatibility, biodegradability, non-toxicity, and cost-effectiveness. Additionally, alginate facilitates mucoadhesion by forming hydrogen bonds with mucin-like glycoproteins, enhancing the formulation’s retention time at the target site [17]. Hydrogels may act as dressings containing intracanal medicaments to eliminate bacteria and control resistance. Interappointment dressings improve root canal disinfection by reducing bacterial presence and inflammation, while also allowing clinicians to monitor treatment progress more effectively [18]. Hydrogels loaded with antimicrobial agents such as chlorhexidine (CHX) have also shown promising endodontic applications. An injectable chlorhexidine-loaded nanotube-modified gelatin methacryloyl hydrogel displayed sustained release of CHX, reduced gelatin degradation, and improved swelling properties [19].

Here, a hydrogel-based dressing containing CUR was developed for PDT targeting endodontic *E. faecalis* infections. The strategy involved the delivery of CUR encapsulated in cetyl palmitate/Tween^®^ 80 solid lipid nanoparticles through an alginate-based hydrogel to ensure CUR’s solubility, stability, and controlled release, aiming to maximize its antimicrobial activity in combination with PDT. This study highlights the potential of this novel formulation as a photoactivated intracanal medicament against *E. faecalis* pathogen.

## 2. Results and Discussion

### 2.1. Characterization of Curcumin-Loaded Nanoparticle-Enriched Hydrogels

#### 2.1.1. Hydrogel Production and Physicochemical Properties

**Figure 1:**
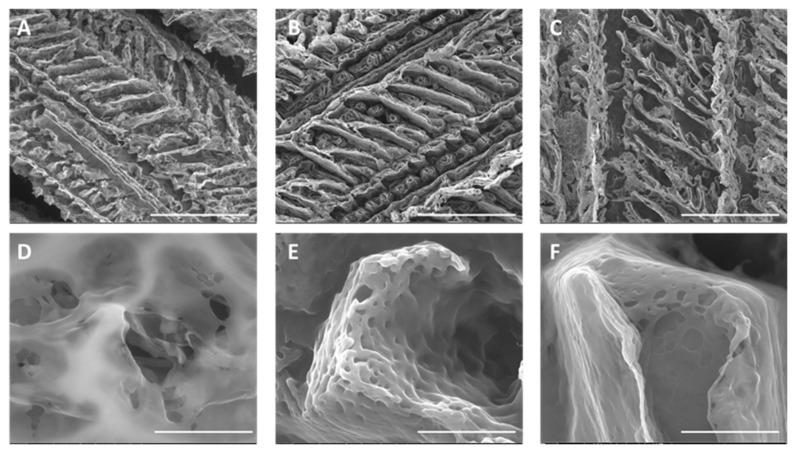
Scanning electron microscopy images of hydrogel morphology. Blank hydrogel (**A**,**D**), SLN-enriched hydrogel (**B**,**E**), and CUR-loaded SLN-enriched hydrogel (**C**,**F**). Scale bar 200 mm (**A**–**C**) and 4 mm (**D**–**F**).

In this study, alginate–PVA hydrogel was synthesized following the methodology established in our previous study [20]. CUR-loaded SLNs of cetyl palmitate-Tween^®^ 80 were prepared as described in [15] and then subsequently incorporated into the hydrogel to serve as an effective vehicle for the intracanal medication. To explore the optimal loading capacity of CUR-loaded SLNs within the hydrogel, two concentrations, 20% and 50% (*v*/*v*), referred to as H-20.NCur and H-50.NCur, were tested. Successful gelation was achieved for both studied dosages. In this study, the alginate–PVA hydrogel was formed through physical interactions, primarily driven by hydrogen bonding and polymer chain entanglement. This gelation process aligns with findings from previous studies [20,21]. The morphology of all produced hydrogels was analyzed by SEM (Figure 1). The blank hydrogel exhibited a highly porous, three-dimensional branched structure, which is well suited for water retention and controlled drug release. Chen et al. observed similar branched porous structures [22]. In this study, the formation of the alginate–PVA hydrogel relies on physical interactions rather than chemical crosslinking. The SEM analysis revealed a highly porous structure in the blank hydrogel, which became denser with reduced pore size upon incorporation of SLNs (Figure 1). This structural modification suggests an increase in polymer network interactions and effective crosslinking density through physical entanglement and hydrogen bonding between alginate, PVA, and nanoparticles. These observations align with the findings of Yu et al. (2024), who reported similar structural modifications in alginate–PVA-based hydrogels, potentially affecting nanoparticle retention and mechanical stability [23].

**Figure 2:**
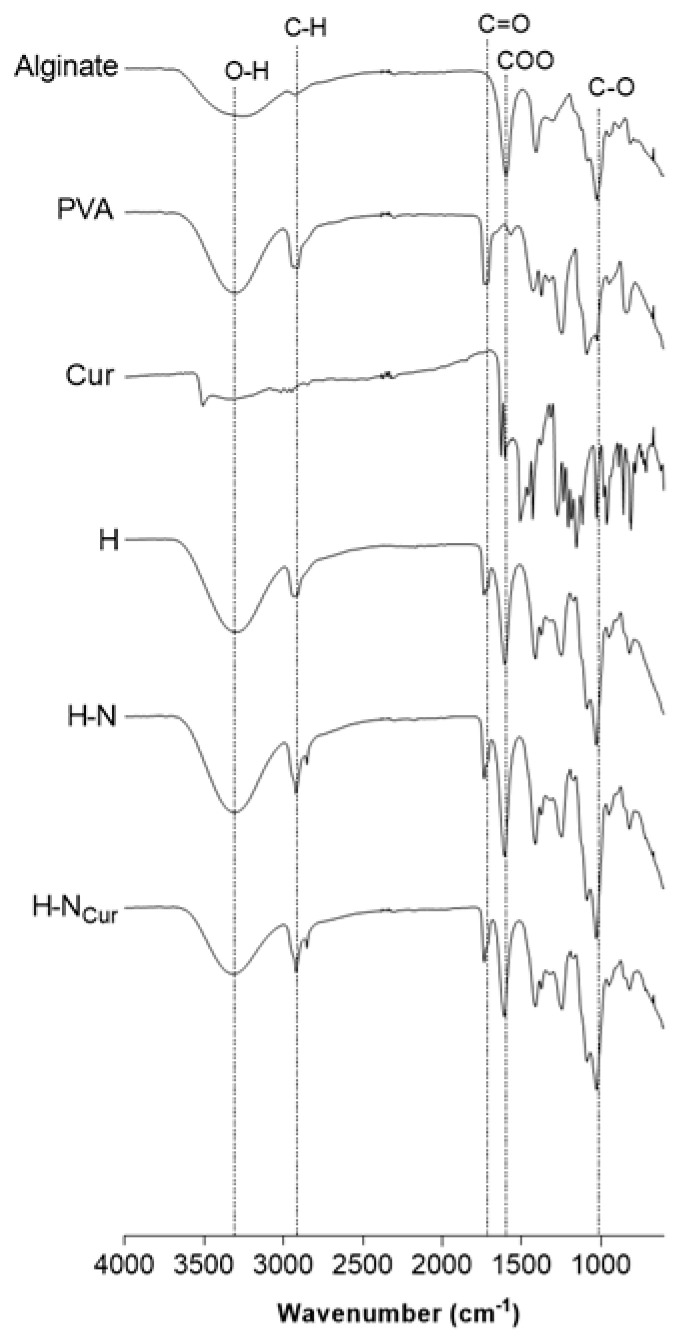
FTIR spectra of alginate, PVA, and curcumin used to produce the hydrogel (H) and the hydrogels enriched with SLNs (H-N) or CUR-loaded SLNs (H-NCur).

FTIR spectroscopy confirmed the successful incorporation of SLNs and CUR within the alginate–PVA gel matrix. The characteristic bands of alginate and PVA, including the -OH stretching (~3300 cm⁻^1^) and carboxylate signals (~1600–1700 cm⁻^1^), were preserved in the hydrogel, indicating that the polymeric matrix remained intact. However, shifts in the C=O stretching region and a slight decrease in the -OH peak intensity were observed in SLN-containing formulations (H-N and H-NCur), suggesting intermolecular interactions between the nanoparticles and the hydrogel network (Figure 2). These findings confirm the role of physical crosslinking in stabilizing the hydrogel matrix and align with Farazin et al. (2021), who demonstrated that nanoparticle incorporation modifies hydrogel network stability through intermolecular interactions, as supported by FTIR analysis [24].

**Figure 3:**
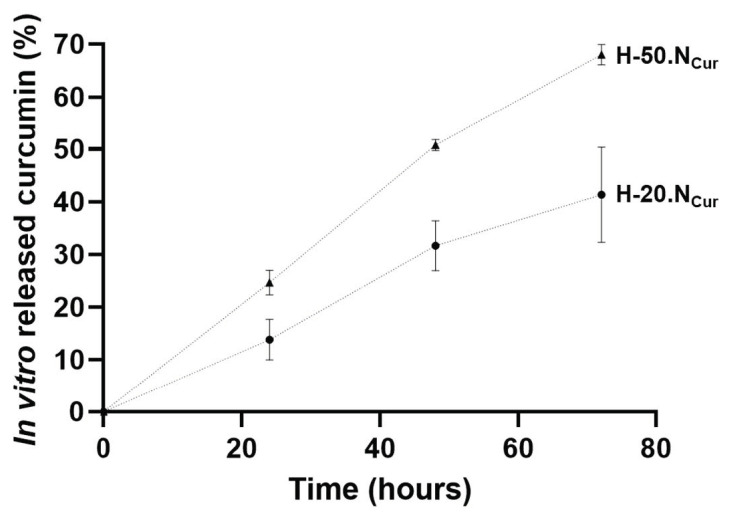
In vitro release profile of curcumin from H-20.NCur and H-50.NCur formulations under physiological pH conditions.

The evaluation of CUR release from the formulations was made in vitro under physiological pH conditions. Both formulations demonstrated a gradual and sustained release over a 72 h period, indicating that the hydrogels provided a controlled CUR delivery system (Figure 3). The absence of an initial burst release suggests efficient encapsulation of CUR within the SLNs and the hydrogel matrix. After 72 h, approximately, 40% and 70% of CUR was released from the H-20.NCur and H-50.NCur formulations, respectively, highlighting their potential for prolonged therapeutic action. Tang and collaborators reported a pH-responsive PVP/CMC hydrogel capable of sustaining the release of 4-aminosalicylic acid, exhibiting also a high drug release capacity (up to 70% in neutral conditions) [25]. The porous structure observed in SEM images (Figure 1) is favorable for water retention, which is critical for controlled drug release. While direct measurements were not performed, the sustained release profile of curcumin over 72 h indirectly supports the hydrogel’s ability to adsorb and retain water within its matrix. The gradual release pattern indicates a steady interaction between the hydrogel matrix and the encapsulated drug, which is influenced by water-uptake dynamics. Similar findings have been reported by Sandhu et al., 2021 [26], where nanoparticle-enriched hydrogels exhibited prolonged release due to their high water-retention capacity.

In vitro drug release data were analyzed according to kinetics models (zero-order, first-order, Korsmeyer–Peppas, Higuchi), and the correlation coefficient values (Table S1) reveal that the formulation demonstrates a zero-order-like release pattern based on the sustained and linear curcumin release kinetics observed in Figure 3. This steady release profile aligns with the evidence of a dual-barrier system: (i) curcumin is entrapped within SLNs, which act as a primary barrier to slow diffusion, and (ii) the alginate–PVA hydrogel matrix provides a secondary barrier, further controlling the release rate through physical entanglement and intermolecular interactions (Figure 1, SEM images). The release profile matches findings by Sandhu et al. (2021), where SLN-hydrogel systems exhibited controlled, zero-order-like release over several days, due to dual-barrier mechanisms [26]. The H-50.NCur formulation released a greater total amount of curcumin, but the release rate remained similar between the two hydrogels. This aligns with the controlled release patterns observed by Yu and collaborators in SLN-enriched hydrogel systems [23]. While not explicitly modeled here, zero-order kinetics are often observed in systems where drug release is governed by polymer erosion or matrix-controlled diffusion rather than concentration gradients. This zero-order-like behavior might ensure prolonged therapeutic activity, critical for intracanal medicaments requiring sustained antimicrobial action.

#### 2.1.2. Hydrogels’ Mechanical Properties

**Figure 4:**
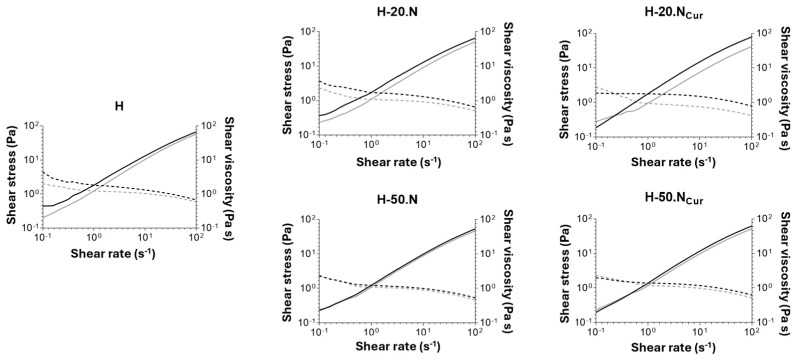
Viscosimetry of freshly prepared (black) and 3-month stored (grey) hydrogels: blank hydrogel (H), hydrogel-enriched with 20% nanoparticles (H-20.N), hydrogel-enriched with 20% CUR-loaded nanoparticles (H-20.NCur), hydrogel-enriched with 50% nanoparticles (H-50.N), and hydrogel-enriched with 50% CUR-loaded nanoparticles (H-50.NCur).

The viscosimetry analysis presented in Figure 4 reveals that all produced hydrogels have similar initial viscosity. Rheological evaluation demonstrated that all hydrogel formulations exhibited shear-thinning behavior, an essential property for injectable biomaterials. The studied formulations display pseudoplastic behavior, as increasing shear rates lead to higher shear stress and reduced shear viscosity. This refers to the property where a viscous formulation becomes less viscous under applied shear stress, such as during syringe-based application with a syringe [27].

A slight decrease in viscosity was observed over the three-month storage period, a phenomenon previously described by Yu et al., who attribute this to potential matrix degradation or molecular rearrangement [23]. The incorporation of CUR-loaded SLNs within the hydrogel did not affect the viscosity, with the exception of H-20.NCur. The presence of nanoparticles at a higher dosage (H-50.N and H-50.NCur) ensures long-term stability of the hydrogel, as no significant effect in viscosity is observed. Similar trends have been documented by Cai and collaborators, suggesting that a higher CUR-loaded SLN dosage enhances the structural integrity of the hydrogel matrix [28].

Rheological analysis reveals that, for all formulations, the initial viscosity (Figure S1) is not fully restored during the recovery period upon application of a high shear stress. Since the initial viscosity is higher than the one observed after the recovering period, the hydrogels exhibit a non-thixotropic profile. This behavior suggests that the hydrogel network undergoes internal structural rearrangements under shear stress, hindering full recovery of its original viscosity. The non-thixotropic nature of these formulations can be attributed to the dynamic interactions between the polymer chains and nanoparticles within the hydrogel matrix. According to Grosskopf et al. polymer–nanoparticle hydrogels exhibit yield stress behavior, with nanoparticles playing a major role in defining the viscoelastic properties and the system’s response to shear [29]. Their study demonstrated that at high shear rates, nanoparticles become immobilized in a compressed state, and network recovery depends on polymer–nanoparticle stoichiometry. In our case, the inability to fully restore viscosity after shear suggests that the shear-induced structural rearrangement alters the polymer–nanoparticle interactions, potentially leading to a partial collapse of the network or reduced entanglement density.

**Figure 5:**
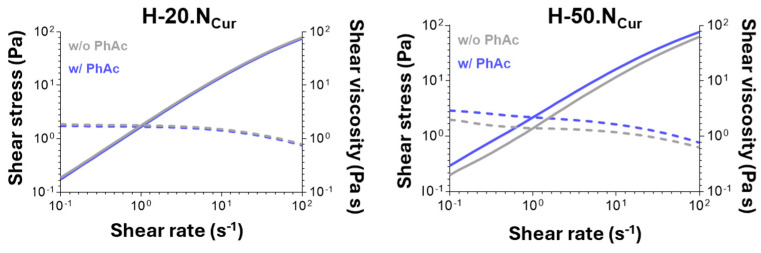
Viscosimetry measurements of hydrogels enriched with 20% (H-20.NCur) or 50% (H-50.NCur) CUR-loaded nanoparticles before (grey) and after (blue) photoactivation.

After 150 s of light exposure, a decrease in viscosity was observed in H-50.NCur formulations (Figure 5), indicating that PDT induces structural modifications in the hydrogel network. This reduction in viscosity could impact the clinical application by potentially influencing the injectability and retention of the hydrogel in the root canal system, highlighting the need for optimization to balance fluidity and structural stability. However, the H-50.NCur formulation retained more viscosity post-PDT compared to H-20.NCur, suggesting that higher CUR-loaded SLN dosage enhances mechanical resistance to PDT-induced changes. Despite the reduction in viscosity, both hydrogels maintained their non-Newtonian shear-thinning properties, ensuring continued injectability following photoactivation. These findings are supported by Willis and coworkers’ report highlighting the role of crosslinking mechanisms in sustaining the rheological stability of curcumin-loaded alginate–PVA hydrogels, enabling structural recovery after exposure to external stimuli [30].

### 2.2. Hydrogel Antimicrobial Activity Mediated by Photoactivation

**Figure 6:**
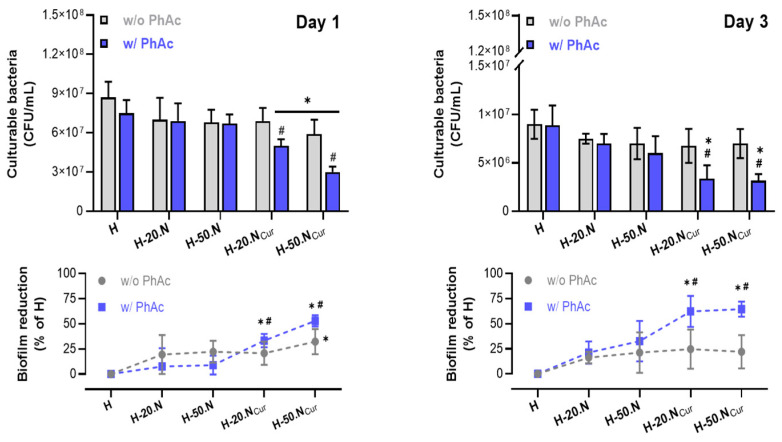
Antibiofilm activity of hydrogel formulations with (w/PhAc) and without (w/o PhAc) photoactivation after 1 and 3 days of culture. * Significantly different from the blank H formulation; # significantly different from the corresponding non-PhAc formulation.

The antibiofilm activity of the developed hydrogel formulations, with and without photoactivation (PhAc), was evaluated over 1 and 3 days. As shown in Figure 6 (top graphs), the presence of CUR in the hydrogel led to a slight reduction in culturable bacteria on day 1 for the formulation H-20.NCur, with a significant reduction observed for H-50.NCur, compared to the blank hydrogel (H). Notably, PhAc-treated CUR-containing formulations exhibited a significantly greater reduction in bacterial load than their non-PhAc counterparts. These results align with Ravazzi et al. (2023), who reported that PDT with CUR-loaded nanoparticles, activated by 450 nm blue LED light, significantly reduced multispecies biofilms in root canals [2]. Additionally, the antimicrobial efficacy of PDT in CUR-containing formulations was corroborated by Pereira et al. (2021), who demonstrated that PDT using a curcumin analog (3,3′-dihydroxycurcumin, DHC) led to a reduction of over 70% in microbial populations within endodontic biofilms [31]. Recent studies also indicate that nanoencapsulation of CUR enhances its antimicrobial activity. Minhaco and co-workers demonstrated that CUR-loaded polymeric nanoparticles activated by blue light result in a significant reduction in endodontic biofilm viability [1], further supporting the findings of the present study.

By day 3, bacterial counts continued to decrease with increasing hydrogel complexity. However, at this time point, only PhAc-H-20.NCur and PhAc-H-50.NCur demonstrated a statistically significant reduction compared to both blank H and their non-PhAc equivalents. In Figure 6 (bottom graphs), where results are normalized to blank H, it is evident that the antibiofilm activity of non-PhAc hydrogels diminishes after 3 days, whereas PhAc-treated CUR-containing formulations maintained a prolonged effect.

**Figure 7:**
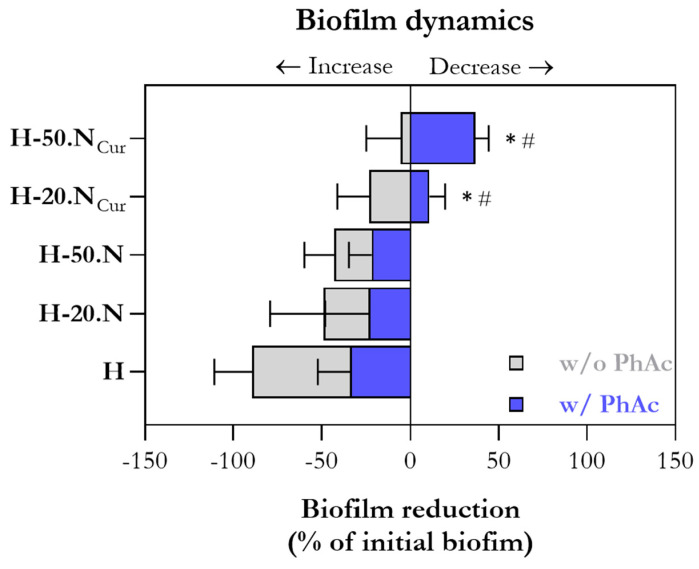
Biofilm reduction relative to the initial biofilm density (time zero) after 1 day of exposure to hydrogel formulations with (w/PhAc) and without (w/o PhAc) photoactivation. * Significantly different from the blank H formulation; # significantly different from the corresponding non-PhAc formulation.

To further investigate biofilm dynamics, biofilm maturation (increase) and biofilm deletion (decrease) were calculated relative to the initial biofilm density at time zero (i.e., immediately before hydrogel application). As Figure 7 illustrates, only PhAc-treated curcumin-containing formulations (H-20.NCur and H-50.NCur) actively inhibited biofilm formation, with the effect being most pronounced in H-50.NCur.

### 2.3. Hydrogel Cytocompatibility Mediated by Photoactivation

**Figure 8:**
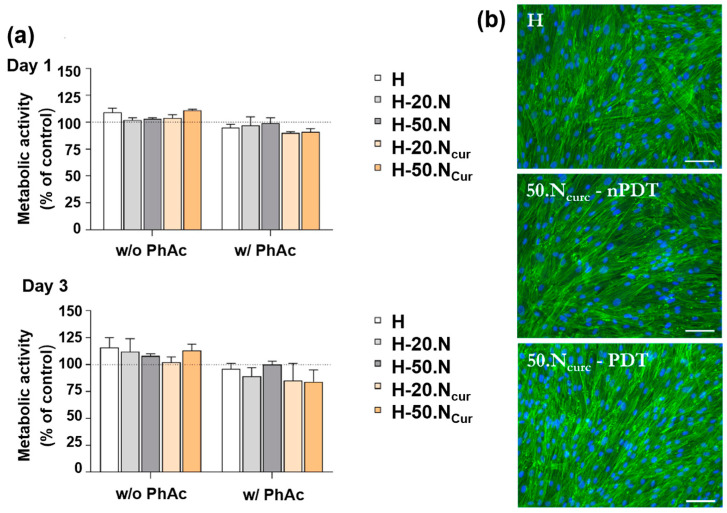
In vitro cytocompatibility of hydrogel formulations with (w/PhAc) and without (w/o PhAc) photoactivation. (**a**) Metabolic activity of hGF, after 1 and 3 days of culture, expressed as a percentage of the control (set at 100%). (**b**) Fluorescence images of hGF after 3 days, showing F-actin cytoskeleton (green) and nuclei (blue) staining. Scale bar: 100 µm.

The cytocompatibility of the hydrogel formulations, with and without PhAc, was evaluated by indirectly exposing hGFs (human gingival fibroblasts) to hydrogels over 1 and 3 days (Figure 8). After 24 h, no adverse effects were observed, with metabolic activity values comparable to the control culture and cultures exposed to blank H (Figure 8a). However, a slight reduction in cell metabolic activity was noted for both PhAc-treated CUR-containing formulations (H-20.NCur and H-50.NCur), a trend that persisted after 3 days. Nevertheless, values were close to 100%. These findings are in line with Sahyon and collaborators, who demonstrated that PDT with CUR activated by blue LED did not significantly reduce fibroblast viability at concentrations below 500 mg/L, reinforcing the safety of CUR in PDT applications [32].

Cell morphology, assessed via immunostaining after 3 days of hydrogel exposure, revealed a dense fibroblast layer with well-defined F-actin striations (Figure 8b), with no observable differences across conditions. These findings support the biocompatibility of nanoencapsulated curcumin, as reported by Bapat et al. (2023), who reviewed its biomedical applications, including its ability to modulate cellular responses without significant toxicity [33].

## 3. Conclusions

This study successfully developed a curcumin-loaded lipid nanoparticle-enriched hydrogel with promising applications for advanced endodontic therapy. The hydrogel demonstrated sustained curcumin release over 72 h, achieving release rates of 40% and 70% for formulations containing 20% and 50% nanoparticles, respectively. Rheological analysis confirmed shear-thinning behavior, ensuring injectability even after photoactivation. Antibiofilm assays revealed significant reductions in *Enterococcus faecalis* biofilms, with photoactivated formulations showing superior efficacy (ca. 70%) compared to non-photoactivated (ca. 45%) counterparts. Cytocompatibility tests verified fibroblast viability across all formulations, supporting their potential for clinical use. These findings highlight the hydrogel’s ability to combine antimicrobial and photodynamic properties, offering an innovative solution to prevent intracanal reinfections in endodontic treatments. Beyond intracanal therapy, this hydrogel system could be adapted for other localized drug delivery applications in dentistry and beyond, including periodontal pockets or post-surgical sites. Further in vivo studies and microbiological models simulating clinical endodontic conditions will be necessary to validate its therapeutic performance and safety.

## 4. Materials and Methods

### 4.1. Materials

Sodium alginate (Lessonia nigrescens, code 177775000, lot A0376873, sulfated ash 34.16% on dried substance) was acquired from ACROS Organics™ (Thermo Fisher Scientific, Waltham, MA, USA). Poly(vinyl alcohol) (PVA) was sourced from Sigma-Aldrich (St. Louis, MO, USA). Cetyl palmitate (Gattefossé, Neuilly-Sur-Seine, France) and Tween^®^ 80 (Sigma-Aldrich, St. Louis, MO, USA) were used in the formulation. Curcumin (CUR) was also obtained from Sigma-Aldrich, while absolute ethanol was purchased from Thermo Fisher Scientific (Waltham, MA, USA). Double-deionized water was prepared using an ultra-pure water system (Arium Pro, Sartorius AG, Gottingen, Germany). The reagents were precisely weighed on a digital analytical balance (Kern ACJ/ACS 80-4, Kern & Sohn, Balingen, Germany). All other reagents were of analytical grade and were used without further purification. For bacterial assays, *Enterococcus faecalis* ATCC 29212 was used as the test strain, with Tryptic Soy Broth (TSB) and Tryptic Soy Agar (TSA) acquired from Liofilchem (Roseto degli Abruzzi, TE, Italy). In cytotoxicity evaluations, human gingival fibroblasts (AG09319) were obtained from the Coriell Institute (Camden, NJ, USA). Cell culture reagents, including α-minimum essential medium (α-MEM), fetal bovine serum (FBS), antibiotic–antimycotic (100×), and phosphate-buffered saline (PBS), were supplied by Gibco (Waltham, MA, USA). Resazurin salt (Sigma-Aldrich, St. Louis, MO, USA) was employed as a viability indicator. Additional reagents such as formaldehyde, Triton X-100, and bovine serum albumin (BSA) were purchased from Sigma-Aldrich (St. Louis, MO, USA). Fluorescent staining agents, including Flash Phalloidin™ Green 488, Hoechst 33342, and Calcein-AM, were provided by BioLegend (San Diego, CA, USA), while propidium iodide (PI) was sourced from BD Biosciences (Franklin Lakes, NJ, USA).

### 4.2. Methods

#### 4.2.1. Preparation of Solid Lipid Nanoparticles

Solid lipid nanoparticles (SLNs), both with and without curcumin (CUR), were produced by the hot ultrasonication method, following the protocol established previously [15]. Briefly, cetyl palmitate (150 mg) was used as the solid lipid, combined with the non-ionic surfactant Tween^®^ 80 (47 mg) as a stabilizing agent and CUR (1 mg) as the bioactive compound. This mixture was heated to 65 °C in a water bath until completely melted, after which 7 mL of pre-warmed water was incorporated. The resulting suspension was subjected to ultrasonic homogenization using a probe-sonicator (VCX130, Sonics & Materials, 115 Newtown, CT, USA) at 70% amplitude for 5 min. Empty SLNs were prepared under identical conditions, excluding the CUR addition.

#### 4.2.2. Preparation of Hydrogels Enriched with Lipid Nanoparticles

The blank hydrogel (H) was obtained by dispersing 0.25 g of sodium alginate in 10 mL of distilled water, followed by vigorous stirring with a spatula in a glass mixing dish until complete dissolution. Subsequently, 5 mL of 10% (*w*/*v*) PVA solution was added, and the mixture was stirred thoroughly until a homogeneous gel was formed (Table 1).

Hydrogels enriched with lipid nanoparticles were prepared by partially replacing the double-deionized water with SLN suspensions obtained in Section 4.2.1. Four enriched hydrogels were obtained: hydrogel containing 20% (*v*/*v*) empty SLN (H-20.N), hydrogel containing 20% (*v*/*v*) CUR-loaded SLN (H-20.NCur), hydrogel containing 50% (*v*/*v*) empty SLN (H-50.N), and hydrogel containing 50% (*v*/*v*) CUR-loaded SLN (H-50.NCur). The preparation followed the same protocol as the blank hydrogel, ensuring homogeneous incorporation of the SLN before adding PVA. All hydrogels were stirred until a uniform consistency was achieved.

#### 4.2.3. Physicochemical Characterization of the Hydrogels

##### Morphological Analysis

To evaluate the morphology of the hydrogels, samples were frozen overnight at −80 °C using a deep freezer (GFL^®^, Burgwedel, Germany) and subsequently lyophilized for 72 h at −80 °C under a vacuum pressure of 0.40 mbar using a freeze dryer (LyoQuest -85 plus v.407, Telstar^®^ Life Science Solutions, Terrassa, Spain). The dried samples were then examined by scanning electron microscopy with a FEI Quanta 400 FEG ESEM/EDAX Pegasus X4M microscope at an accelerating voltage of 10 kV. Before imaging, the hydrogels were mounted on carbon-taped metal stubs and coated with a thin Au/Pd layer via sputtering for 45 s to enhance conductivity.

##### Rheology Studies

The rheological properties of the hydrogels were assessed using a rheometer (Malvern Kinexus Lab+; Malvern Instruments; Worcestershire, UK) through two different methodologies. Viscosity measurements were performed using a shear rate table method (0.1 to 100.0 s⁻^1^, 10 samples per decade, 25 °C). Thixotropy behavior was analyzed through a three-step shear rate test: an initial phase at 0.1 s⁻^1^ for 2 min, followed by a high shear phase at 100.0 s⁻^1^ for 30 s and a recovery phase at 0.1 s⁻^1^ for 15 min, all conducted at 25 °C. A plate–plate geometry (CP4/40 SR4321) with a 1 mm gap (Peltier Plate Cartridge) was used for all experiments. Each measurement was performed in triplicate, and data were collected using the rSpace software (Kinexus 1.75: PSS0211-17). For the photodynamic activation (PDT) effect on the viscosity of the CUR-enriched hydrogels, a 150 s irradiation was performed with an LED curing light (430-490 nm, SPEC3, Coltene, Altstatten, Switzerland) before starting viscosimetry analysis as described above.

To evaluate the stability of the formulations over time, rheological analyses were performed on all hydrogel samples (H, H-20.N, H-20.NCur, H-50.N, H-50.NCur). These analyses were conducted immediately after preparation (T0m—freshly prepared) and after three months (T3m) of storage at room temperature in sealed vials protected from light. The assessment focused on potential changes in viscosity and thixotropy, providing insights into formulation stability under storage conditions.

##### Chemical Interactions Analysis by Fourier Transform Infrared Spectroscopy

Following the lyophilization process (described above), the hydrogels were analyzed using Fourier-transform infrared (FTIR) spectroscopy with a Frontier™ spectrophotometer (PerkinElmer, Santa Clara, CA, USA) equipped with a diamond crystal. The produced formulation and individual components were analyzed directly on the ATR compartment at room temperature. A background scan was performed with an empty ATR compartment to eliminate potential interferences. The spectra were obtained by averaging 32 scans recorded in the range of 4000 to 600 cm⁻^1^, with a spectral resolution of 4 cm⁻^1^.

#### 4.2.4. In Vitro Curcumin Release from the Hydrogels

The in vitro release profile of CUR from the SLNs embedded within the hydrogel was achieved using the Slide-A-Lyzer^®^ MINI Dialysis Devices, 10K MWCO (Thermo Scientific). Thus, 0.5 mL of H-20.NCur and H-50.NCur containing 9.5 or 24 μg of CUR, respectively, were transferred into the cup device before submersion in the conical tube containing 14 mL of release medium (PBS with 10% ethanol to achieve sink conditions) for 72 h. At predetermined intervals of 24, 48, and 72 h, 1 mL samples were collected and replaced with an equal volume of fresh release medium. The devices were kept in an orbital shaker (ES-60E Incubater Shaker, Miulab, Hangzhou, Zhejiang, China) at 37 ± 0.5 °C, under constant agitation of 100 rpm. The amount of CUR released was spectrophotometrically determined at 430 nm by a Jasco V-660 Spectrophotometer (Piscataway, NJ, USA). The assay was carried out in triplicate. To determine the mechanism of release, the experimental data were fitted with several commonly used kinetics models (zero-order, first-order, Korsmeyer-Peppas, Higuchi) of the drug release process, as previously described [15].

#### 4.2.5. Antibiofilm Activity

A bacterial suspension of *Enterococcus faecalis* ATCC 29212 was prepared in TSB and adjusted to a concentration of 10^8^ colony-forming units (CFU)/mL. This suspension was transferred to the wells of 24-well plates and incubated at 37 °C and 120 rpm for 24 h to allow biofilm formation. After incubation, the wells were washed with 0.9% NaCl solution to remove non-adhered bacteria. Hydrogel formulations (200 µL per well) were then applied. For each condition, half of the replicas (n = 3) were not subjected to further treatment (w/o PhAc), while the other half (n = 3) were irradiated with an LED curing light (430–490 nm, SPEC3, Coltene, Altstatten, Switzerland) for 150 s (w/PhAc). The plates were then incubated for an additional 24 h at 37 °C. To quantify the remaining biofilm, the wells were sonicated in an ultrasonic water bath (35 kHz, Sonorex, Bandelin, Berlin, Germany) for 15 min to dislodge sessile microorganisms. Aliquots from each well were serially diluted 10-fold, plated on TSA plates, and incubated at 37 °C for 24 h to determine the CFU counts. Three independent experiments were conducted. The results were expressed as CFUs/mL, and as the percentage of biofilm reduction, measured relative to blank hydrogel (H) and to the initial biofilm density at time zero (i.e., immediately before the addition of hydrogel formulations).

#### 4.2.6. In Vitro Cytocompatibility

##### Cell Culture Conditions

Human gingival fibroblasts (hGFs) were cultured in α-MEM supplemented with 10% fetal bovine serum and 1% antibiotic-antimycotic solution at 37 °C in a humidified atmosphere with 5% CO2. hGFs were seeded at 10^4^ cells/cm^2^ into 24-well plates and allowed to adhere for 24 h. Hydrogel formulations (200 µL) were placed in culture inserts (0.4 µm pore, Greiner Bio-One, Monroe, NC, USA). For each formulation, half of the replicas (n = 3) were irradiated with an LED curing light (430–490 nm, SPEC3, Coltene, Altstatten, Switzerland) for 150 s (w/PhAc), while the remaining replicates (n = 3) were not irradiated (w/o PhAC). The inserts were placed in the wells containing the cultured cells, and the system was incubated for 3 days. Positive control cultures were maintained without hydrogel exposure. The cultures were assessed for metabolic activity and cell morphology.

##### Metabolic Activity

After 1 and 3 days, cell metabolic activity was evaluated by resazurin assay. Inserts were temporarily removed, and the medium was replaced with fresh medium containing 10% resazurin (0.1 mg/mL). After 3 h of incubation at 37 °C, fluorescence was measured using a microplate reader (Synergy HT, Biotek, Santa Clara, CA, USA) at excitation/emission wavelengths of 530/590 nm. Results were plotted relative to the positive control cultures.

##### Cell Morphology

After 3 days, cell morphology was evaluated after immunostaining for filamentous actin (F-actin) and nuclei. Cell monolayers were fixed with 3.7% formaldehyde for 15 min, permeabilized with 0.1% Triton X-100 for 15 min, and blocked with 1% BSA for 30 min. F-actin was stained with Flash Phalloidin 488 (1:200) for 30 min, and the nuclei were counterstained with Hoechst 33342 (10 μg/mL) for 10 min. A Celena S digital imaging system (Logos Biosystems, Anyang-si, Republic of Korea) was used for visualization.

#### 4.2.7. Statistical Analysis

Data were plotted as mean ± standard deviation. Statistical significance between groups was assessed using one-way ANOVA followed by Tukey’s post hoc test for multiple comparisons (SPSS software, version 28.0, IBM^®^). A *p*-value < 0.05 was considered statistically significant.

## Figures and Tables

**Table 1:** Composition of hydrogel formulations enriched with lipid nanoparticles.

Hydrogel Code/Ingredient	Sodium Alginate	PVA 10% (*w*/*v*)	Water (mL)	Empty SLN (mL)	CUR-Loaded SLN (mL)
H	0.25 g	5 mL	10 mL	–	–
H-20.N	0.25 g	5 mL	8 mL	2 mL	–
H-50.N	0.25 g	5 mL	5 mL	5 mL	–
H-20.NCur	0.25 g	5 mL	8 mL	–	2 mL
H-50.NCur	0.25 g	5 mL	5 mL	–	5 mL

SLN, solid lipid nanoparticles; CUR-loaded SLN, curcumin-loaded solid lipid nanoparticles; PVA, polyvinyl alcohol.

## Data Availability

Data are contained within the article.

## Supplementary Materials

The following supporting information can be downloaded at https://www.mdpi.com/article/10.3390/gels11050308/s1, Figure S1: Thixotropy analysis of blank hydrogel (H), hydrogel-enriched with 20% nanoparticles (H-20.N), hydrogel-enriched with 20% CUR-loaded nanoparticles (H-20.N_Cur_), hydrogel-enriched with 50% nanoparticles (H-50.N), hydrogel-enriched with 50% CUR-loaded nanoparticles (H-50.N_Cur_); Table S1: Release kinetic parameters (r2) for CUR loaded-SLN in hydrogel data obtained using several mathematical models.

## Abbreviations

The following abbreviations are used in this manuscript:

α-MEM	α-minimum essential medium
BSA	bovine serum albumin
CHX	chlorhexidine
CFU	colony-forming units
CUR	curcumin
*E. faecalis*	*Enterococcus faecalis*
FBS	fetal bovine serum
f-actin	filamentous actin
FTIR	Fourier-transform infrared spectroscopy
hGF	Human gingival fibroblasts
H	hydrogel
H-20.N	hydrogel containing 20% (*v*/*v*) empty SLN
H-50.N	hydrogel containing 50% (*v*/*v*) empty SLN
H-20.NCur	hydrogel containing 20% (*v*/*v*) CUR-loaded SLN
H-50.NCur	hydrogel containing 50% (*v*/*v*) CUR-loaded SLN
Non-PhAc	non-photoactivated
PBS	phosphate-buffered saline
PDT	photodynamic therapy
PhAc	photoactivated
PI	propidium iodide
PVA	poly(vinyl alcohol)
ROS	reactive oxygen species
SEM	scanning electron microscopy
SLNs	solid lipid nanoparticles
TSA	Tryptic Soy Agar
TSB	Tryptic Soy Broth
