# Supplementary material for: Photoactivated Curcumin-Loaded Lipid Nanoparticles in Hydrogel: A Cutting-Edge Intracanal Medicament for Advanced Endodontic Therapy
Source: Gels. 2025 Apr 22;11(5):308. doi: 10.3390/gels11050308 (PMC12111544; doi:10.3390/gels11050308)
Supplement: Supplementary file 1 [file gels-11-00308-s001.zip › gels-3566116-supplementary.pdf]

# Photoactivated Curcumin-Loaded Lipid Nanoparticles in Hydrogel: A Cutting-Edge Intracanal Medicament for Advanced Endodontic Therapy

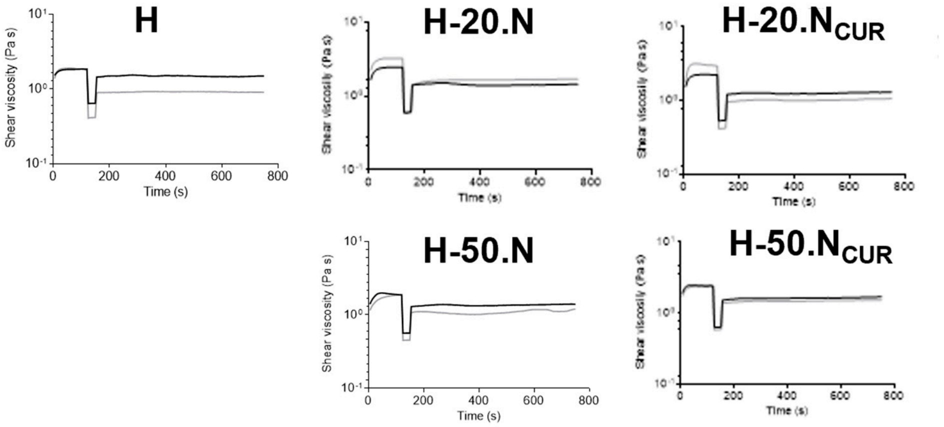


**Figure S1.** Thixotropy analysis of blank hydrogel (H), hydrogel-enriched with 20% nanoparticles (H-20.N), hydrogel-enriched with 20% CUR-loaded nanoparticles (H-20.$N_{Cur}$), hydrogel-enriched with 50% nanoparticles (H-50.N), hydrogel-enriched with 50% CUR-loaded nanoparticles (H-50.$N_{Cur}$).

**Table S1.** Release kinetic parameters (r2) for CUR loaded-SLN in hydrogel data obtained using several mathematical models

| Release model | $r^2$ |
|---|---|
| **Zero order** | **0.995** |
| First order | 0.989 |
| Higuchi | 0.749 |
| Korsmeyer-Peppas | 0.841 |
